# Maternal Regulation of Pups’ Cortical Activity: Role of Serotonergic Signaling

**DOI:** 10.1523/ENEURO.0093-18.2018

**Published:** 2018-07-31

**Authors:** Emmanuelle Courtiol, Donald A. Wilson, Relish Shah, Regina M. Sullivan, Catia M. Teixeira

**Affiliations:** 1 Emotional Brain Institute, Nathan Kline Institute for Psychiatric Research, Orangeburg, NY 10962; 2 Department of Child and Adolescent Psychiatry, New York University School of Medicine, New York, NY 10016

**Keywords:** serotonin, rat pups, maternal interaction, prefrontal cortex, oscillation

## Abstract

A developing brain shows intense reorganization and heightened neuronal plasticity allowing for environmental modulation of its development. During early life, maternal care is a key factor of this environment and defects in this care can derail adaptive brain development and may result in susceptibility to neuropsychiatric disorders. Nevertheless, the mechanisms by which those maternal interactions immediately impact the offspring’s brain activity to initiate the pathway to pathology are not well understood. We do know that multiple neurotransmitter systems are involved, including the serotonergic system, a key neuromodulator involved in brain development and emotional regulation. We tested the importance of the serotonergic system and pups’ immediate neural response to maternal presence using wireless electrophysiological recordings, a novel approach allowing us to record neural activity during pups’ interactions with their mother. We found that maternal contact modulates the P10–P12 rat pups’ anterior cingulate cortex (ACC) activity by notably increasing local-field potential (LFP) power in low-frequency bands. We demonstrated, by blocking serotonergic receptors, that this increase is mediated through 5-HT2 receptors (5-HT2Rs). Finally, we showed in isolated pups that enhancing serotonergic transmission, using a selective-serotonin-reuptake-inhibitor, is sufficient to enhance LFP power in low-frequency bands in a pattern similar to that observed when the mother is in the nest. Our results highlight a significant contribution of the serotonergic system in mediating changes of cortical activity in pups related to maternal presence.

## Visual Abstract

**Figure:**
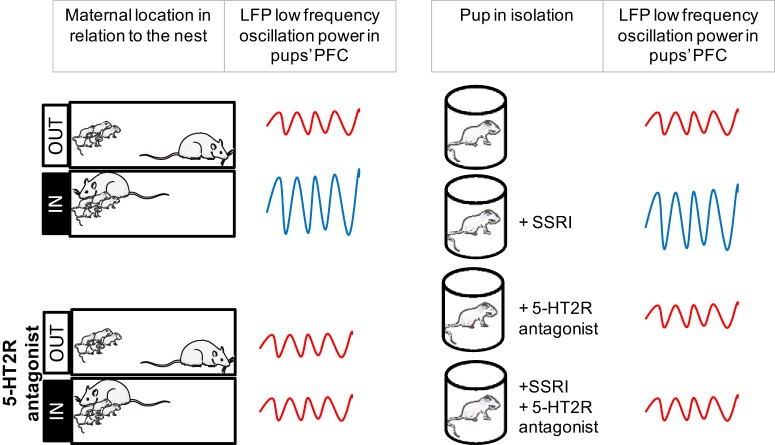

## Significance Statement

Maternal care is a major environmental factor influencing brain development, and deficits in this care can result in maladaptive behaviors in the offspring. While maternal interaction is crucial, little is known about the underlying mechanisms by which it modulates infant/pup brain activity. In this study, using wireless electrophysiological recordings, we show that the mother’s contact regulates the activity of key prefrontal regions of 10- to 12-d-old rat pups. Regarding mechanisms, we found that the serotonergic system contributes to this modulation, notably through 5-HT2 receptors (5-HT2Rs). These results suggest maternal care affects serotonergic neural activity during early life and provide key insights into how maternal care affects the adaptive/maladaptive development of brain circuits implicated in adult pathology.

## Introduction

The postnatal period is a critical stage during which the brain undergoes major changes: new wiring, organization, and intense plasticity. This synaptic plasticity serves a dual function of organizing the infant’s immediate behavior, while programming the brain for future adaptive behaviors. Thus, any environmental factor that can affect the developing brain during this period can have long-lasting repercussions (Chaudhury et al., 2016). At this early stage of life, one of the major environmental inputs is maternal care. Maternal presence, the quality of maternal care, and the removal of that care during prolonged separation during early-life has been shown, in humans and rodents alike, to regulate fear (Routh et al., 1978; Barr et al., 2009), to mediate life-long changes in mood (Vetulani, 2013), to modulate response to stress (Hellstrom et al., 2012; Rincón-Cortés et al., 2015), and influence drug consumption (Delavari et al., 2016). Maternal interactions during early-life are thus critical for individuals’ life-long mental health (Nelson et al., 2011; Cameron et al., 2017; Sale, 2018). Recently, pup cortical activity has been found to be influenced by maternal interactions. Using local-field potential (LFP) recordings in behaving rat pups (P12–P19), maternal presence was found to be related to an increase in low-frequency neural activity, and norepinephrine was found to mediate the pup’s neural responses to maternal milk ejection (Sarro et al., 2014). While norepinephrine can mediate the pup’s neural responses to milk ejection, the exact mechanisms by which maternal presence/absence regulates the developing brain’s activity are not yet understood. Considering that maternal neglect is the most prevalent form of child abuse, with important societal consequences (U.S. Department of Health and Human Services 2010), it is critical to dissect and understand these mechanisms.

Serotonin (5-HT) is a key neuromodulator known to regulate expression of maternal behaviors (Angoa-Pérez et al., 2014). In developing rodents, serotonin levels peak during the perinatal phase (Hohmann et al., 1988). Importantly, modifying serotonergic signaling during early-life leads to changes in cortical pyramidal neuron morphology as well as emotional deficits in adulthood (Ansorge et al., 2004, 2008; Rebello et al., 2014). Those deficits appear to be mediated through 5-HT2 receptors (5-HT2Rs; Sarkar et al., 2014). One of the highest density of serotonergic terminals in the neocortex is found in the prefrontal cortex (PFC), where multiple serotonergic receptors, particularly 5-HT1A and 5-HT2R, are abundant (Celada et al., 2013). Importantly, in the developing PFC, there is a dramatic shift in 5-HT receptor function and expression (Beique et al., 2004). Beique et al. (2004) show that in slices of P6–P19 rats, 5-HT elicits depolarization of pyramidal neurons that shifts to hyperpolarization starting in the third postnatal week. A change in receptor expression occurs with a decrease in 5-HT7R-mediated depolarization and the appearance of 5-HT1AR-mediated hyperpolarization. At the same time, shifts in the effectiveness, rather than shifts in receptor expression, are associated with a decline in 5-HT2AR-mediated depolarization (Beique et al., 2004). The PFC is a critical structure subserving various cognitive functions (Bechara et al., 1998; Miller and Cohen, 2001; Schoenbaum et al., 2002), including behavioral flexibility and working memory (Tottenham et al., 2011; Parnaudeau et al., 2013; Funahashi, 2017). The PFC has a prolonged developmental period, extending high levels of plasticity into adolescence, which allows it to adapt to environmental changes but also makes it more susceptible to environmental insults that can lead to maladaptive development and consequent behavioral deficits (Kolb et al., 2012). Serotonin is thus a good candidate to participate in the regulation of developing brain activity, especially PFC activity, associated with maternal care.

Using wireless LFP recording, we recorded pups’ PFC [specifically in the anterior cingulate cortex (ACC) region] activity in their home cage with littermates and dam. We found that the dam’s presence and absence from the nest modulated the PFC activity of rat pups. First, we found that LFP power was increased in low-frequency bands during periods when the dam was in contact with the pup. Second, we found that blocking 5-HT2R blocked the increase in low-frequency oscillations associated with the maternal contact. Third, we observed that increasing serotonergic signaling, using a selective-serotonin-reuptake-inhibitor (SSRI), was sufficient to increase LFP power in low-frequency bands compared to controls and that this increase was mediated through 5-HT2R. Our results reveal the contribution of the serotonergic system in the regulation of pups’ cortical activity in response to maternal interactions.

## Materials and Methods

### Animals

Long Evans rats (dam and pups) were used for these experiments (Taconic Farms). Twenty-four male rat pups (P9–P12) from 12 litters were used for the LFP experiments, 12 male pups (P11–P16) from six litters were used for the nipple-attachment experiment, and 24 pups (P7–P12) from six litters were used for the ultrasonic vocalizations (USVs) experiment. The pups were bred in our colony and were maintained on a 12/12 h light/dark cycle with access to food and water *ad libitum*. For the LFP experiments, litters were culled to four to six pups and habituated to the cage and experimental room for 3 d before surgery to ensure a calm dam during recordings. Surgeries were performed on P9–P11 rat pups and recordings were performed the day after surgery (P10–P12). This range of ages was chosen because maternal regulation of pups’ brain activity is maximal before P16 (Sarro et al., 2014), and P9 was the earliest time at which this surgery could be reliably performed by us.

Experiments were conducted blind to pup treatment condition. All animal procedures were performed in accordance with the local animal care committee’s regulations and NIH guidelines.

### Surgery for LFP recording

**Figure 1.**
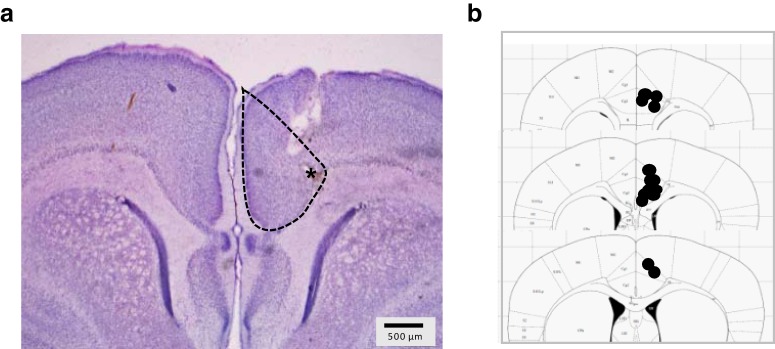
Electrode placement. ***A***, Illustrative cresyl violet staining of a pup brain showing electrode track marks. ***B***, The electrodes were placed in the ACC area of the PFC.

Under isoflurane anesthesia (5% for induction and 1.5–3% for maintenance), a stainless-steel electrode (A-M Systems) was implanted in the ACC region of the PFC (AP: +2.0 mm, ML: -0.5 mm, DV: -2 mm; Fig. 1*A*,*B*) and cemented to the skull. The electrode was previously connected to a telemetry device (ETA-F10, DSI). The telemetry device was placed subcutaneously under the dorsal skin. The incision was closed using surgical suture (Stoelting) and the area of the incision was cleaned using sterile water to remove as much extraneous odors as possible. After surgery, the pups were kept warm until fully recovered from anesthesia (**∼**2 min). The pups were then gently covered with some used bedding and placed back in the nest. Surgeries were performed in two male pups per litter.

### Drugs

To test the contribution of the serotonergic system on pups’ neural activity associated with maternal presence, pharmacological modifications were performed using drugs injected intraperitoneally.

To block serotonergic signaling, we targeted 5-HT2R using the 5-HT_2A/2C_R antagonist ketanserin (2.5 mg/kg; Tocris, catalog #0908; Sarkar et al., 2014). Ketanserin is a strong 5-HT2AR and 5-HT2CR antagonist with weak associated α‐adrenergic blocking and anti-histamine activity (Awouters, 1985). To enhance serotonergic signaling, we used the SSRI fluoxetine (10 mg/kg; Sigma, catalog #1279804; Ansorge et al., 2004). These drugs and vehicle (0.9% saline) were injected at a volume of 0.02 ml per 10 g. Fluoxetine does not readily dissolve in saline solution so fluoxetine was first diluted in sterile water and then NaCl was added. Average pup weight was 25.46 ± 4.7 g. To ensure that the experimenter was blind to the treatment of the animals, the solutions were prepared by a different experimenter and color coded. The color code was only revealed after histologic and data analyses.

### Experimental design (LFP recordings)

Twenty-four hours following surgery, pups’ neural activity was recorded during the different stages of the behavioral experiments. LFP signals were acquired, digitized and stored using Spike 2 software (CED). Twenty-four male pups were implanted and recorded for these experiments (two were excluded due to frequent signal artifacts). Two pups were recorded per day, one in the morning and one in the afternoon. The drug treatment administered in the morning or the afternoon was counterbalanced. The wireless LFP recording approach chosen allows recording during undisturbed maternal interactions in the home cage. Neural activity of P10–P12 pups was recorded in two housing setups: in the home cage with the dam and littermates present, and in isolation in a 500-ml warm plastic beaker. The home cage (47 × 25 cm) was covered with a layer of pine shaving bedding and included a plastic hutch under which the nest was generally located. During home cage periods, the presence (in nest) and absence (out of nest) of the dam from the nest were observed directly and time stamped onto the LFP recording file. “In nest” behavior was recorded as the time when the dam was touching the nest. “Out of nest” was recorded when the dam was not in physical contact with the nest. During this period, the motorically immature pups mainly remained huddled in the nest, while the dams, engaged in eating/drinking, roaming the cage and resting either on top of the hutch or elsewhere in the cage. Maternal in nest and out of nest transitions were not analyzed to ensure uninterrupted and consistent recordings related to maternal presence or absence.

Three experiments were performed on each pup in a serial sequence:

#### Experiment 1

**Figure 2.**
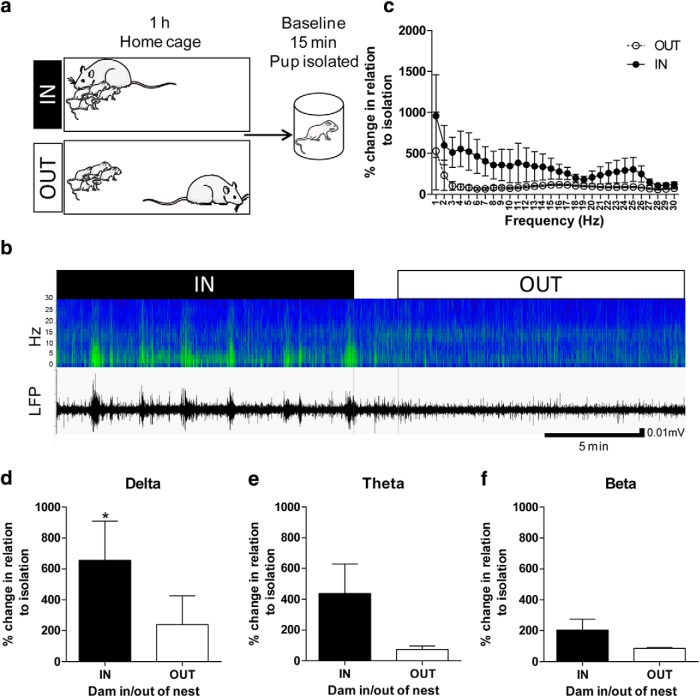
Maternal presence increases low-frequency oscillation power in pups’ brains. ***A***, Experimental design. Rat pups were implanted in the PFC with a wireless LFP recording device. The next day, LFPs were recorded during home cage activity (dam in the nest area: IN; dam in the cage but outside the nest area: OUT) and isolation. The IN and OUT data were normalized in respect to when the pup was in isolation (maternal location/isolation × 100). ***B***, Example of sonogram (top) and LFP (bottom) recorded in one pup within the same session. IN represents the period when the dam was in the nest and OUT represents the period when the dam was out of the nest. ***C***, Maternal presence in the nest tended to increase LFP power across most of the frequencies recorded (0–30 Hz). ***D***, δ power was significantly increased when the dam was present in the nest. ***E***, There was a tendency toward increased LFP power in the θ range during maternal presence. ***F***, β oscillation power was not significantly affected by maternal presence. *N* = 13; *p < 0.05. Images of rat and young rat, Servier Medical Art by Servier is licensed under a Creative Commons Attribution 3.0 Unported License (Figs. 2–4 and visual abstract).

LFP recordings were obtained during an hour in the home cage during which the presence or absence of the dam in the nest was time stamped. After this period, the pups were placed in isolation in a beaker containing home cage bedding for 15 min (Fig. 2*A*). This period of isolation was used as baseline for data analysis. Within the ∼1-h homecage recording, LFP signals were only analyzed in pups in which we obtained uninterrupted periods of presence or absence of the dam in the nest of at least 5 min.

#### Experiment 2

After experiment 1, pups were removed from the litter, weighed, injected with ketanserin or vehicle, and immediately placed back in the nest. This procedure took <1 min. Pup treatment order was alternated between litters, so half the time the first pup recorded received saline and the other half, ketanserin.

**Figure 3.**
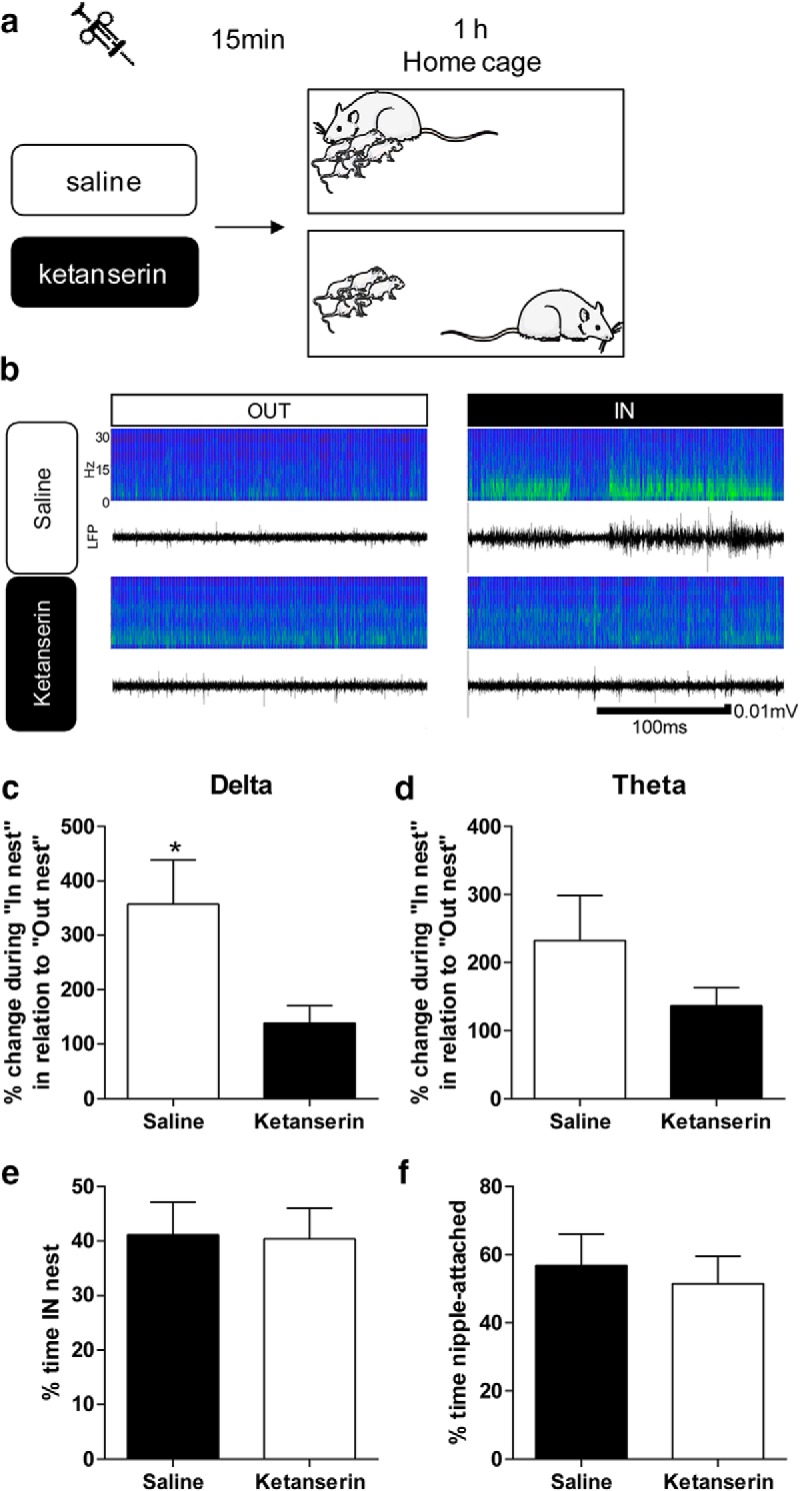
Ketanserin blocks maternal regulation of pups’ brain activity. ***A***, Experimental design. ***B***, Example of sonograms and LFPs recorded within the same session during In and Out of the nest period for a pup injected with saline (top) and another pup injected with ketanserin (bottom). ***C***, Ketanserin blocked the effect of maternal presence (ratio of “In nest”/“Out of nest”*100) on pups’ PFC activity in the δ frequency band compared to saline injection. ***D***, There was no significant difference between saline and ketanserin treated animals in the θ band. *N* = 6 ketanserin; *N* = 7 saline. ***E***, There were no differences in the percentage of time spent in the nest with the mother in the saline and ketanserin recordings. ***F***, In a separate group of animals, the time spent nipple-attached was quantified. There were no differences between saline and ketanserin injected animals (*N* = 6 per group). * *p* < 0.05.

Fifteen minutes after the injection, LFP recording was started and the presence/absence of the dam in the nest was registered and time stamped onto the recording file (Fig. 3*A*). Within the **∼**1 h of recording, LFP signals were only analyzed in pups in which we obtained uninterrupted period of presence or absence of the dam in the nest of at least 5 min.

#### Experiment 3

**Figure 4.**
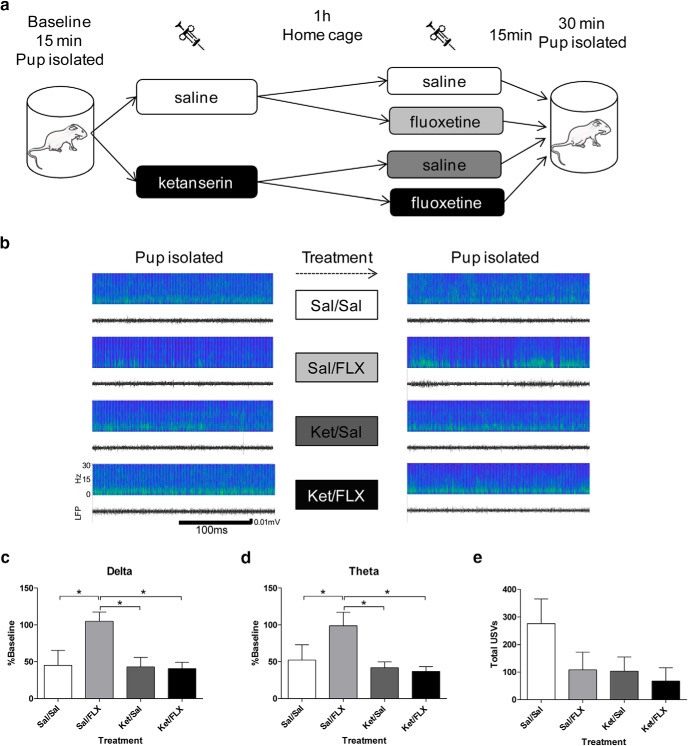
Fluoxetine administration increases LFP power in the low-frequency bands during isolation compared to controls. ***A***, Experimental design. An implanted pup was placed in isolation for 15 min while baseline LFP was recorded. The pup was injected with ketanserin or saline for the first injection and returned to their home cage. One hour later, the pup was injected with fluoxetine or saline. The pup was then placed in isolation and 15 min later, the LFPs were recorded for 30 min. ***B***, Example of sonograms and LFPs recorded within the same session during the first (left) and second (right, after the treatments) isolated periods in pups injected (from top to bottom) with Sal/Sal, Sal/FLX, Ket/Sal, and Ket/FLX. ***C–D***, Fluoxetine administration increased LFP power in δ and θ bands during isolation. This effect was blocked by the 5-HT2R antagonist ketanserin *N* = 4 in each group. ***E***, USVs in isolated pups (*N* = 6 per group). Sal = saline, Ket = ketanserin, FLX = fluoxetine. * *p* < 0.05.

Following the ketanserin or saline injection and subsequent **∼**1 h home cage period of experiment 2, pups were subdivided to receive a second drug administration of fluoxetine or vehicle, in a pseudo-random order, to produce four treatment groups: saline-saline, saline-fluoxetine, ketanserin-saline, and ketanserin-fluoxetine. Immediately after the second injection, pups were placed in isolation and after a 15-min wait period, LFPs were recorded for 30 min (Fig. 4*A*).

### Experimental design (nipple-attachment)

To test whether ketanserin affects the behavior of the pups in the nest we assessed nursing behavior. Two male pups (P11–P16) per litter, from six litters, were randomly selected and one was injected with saline and one with ketanserin. The pups were returned to the home cage with only the mother present. In this setup, all the other littermates and the plastic hutch were removed two hours before the testing to facilitate observation of nursing. Thirty minutes after injection, the duration each pup was nipple-attached was recorded by an observer.

### Experimental design (USVs)

Four male pups (P7–P12) per litter were randomly selected and injected with saline or ketanserin and returned to their home cage for 1 h. They were then injected with saline or fluoxetine and placed back in their home cage to produce four treatment groups: saline-saline, saline-fluoxetine, ketanserin-saline, and ketanserin-fluoxetine. Six litters were tested in this experiment. Thirty minutes later, the pups were removed from the litter and placed in isolation in a beaker, positioned under a ULTRAMIC200K (Dodotronic) microphone, inside a sound proof box. USVs were recorded for 30 min and analyzed using the SeaWave (CIPRA and AEST) software. The number of vocalizations in frequencies between 30 and 65 Hz (Simola, 2015) was quantified.

### LFP analysis

LFP signals were analyzed offline using Spike 2 during the different behavioral epochs. Fast Fourier transform (FFT) power analyses were performed over periods of several minutes (defined by the markers for each behavioral period) using a Hanning window with 0.9766-Hz frequency bins. To correct for interanimal variability, LFP power was expressed as a ratio relative to the first period when the pup was isolated in the beaker (experiments 1 and 3, maternal location/isolation*100 and treatment/isolation*100, respectively) or relative to the out of nest period (experiment 2, in nest/out of nest*100). We then calculated the average power in the different frequency bands: δ (0–3.9 Hz), θ (3.9–7.8 Hz), and β (15.6–31.3 Hz).

### Statistical analysis

Statistical analysis was performed using GraphPad Prism software with α of 0.05. To test differences between groups across frequencies, we used two-way repeated measures (RM) ANOVA. To compare the normalized LFP power between in nest and out nest conditions within animals in experiment 1, we performed paired *t* tests. To test for differences between treatment groups composed of independent animals in experiment 2, we used unpaired *t* tests. To test for differences between treatment groups in experiment 3, we used one-way ANOVA followed by LSD *post hoc* test. Data are reported as mean ± SEM.

### Anatomic location of electrode placement

At the completion of the experiments, pups were overdosed with urethane (1.25 g/kg), brains were removed, fixed, and cryoprotected in a 4% PFA + 30% sucrose solution. Coronal brain sections (40–50 μm thick) were cut on a microtome (Leica). Sections were mounted on slides, stained with cresyl violet, and electrode placements were examined under a light microscope. Electrode placement predominantly targeted the ACC portion of the PFC (Schweimer and Hauber, 2005; Fig. 1*A*,*B*). The tracks of some electrodes passed through the motor cortex, with some electrode tips being located close to the motor cortex. Six pups in which the electrode tracks showed damage to the corpus callosum were removed from analyses.

## Results

### Maternal presence/absence from the nest affects PFC activity in pups

Male rat pups (P10 ± 1 d) were implanted with an electrode targeted to the ACC region of the PFC and connected to a telemetry device.

The day after surgery, cortical LFPs were recorded in the pups’ home cage for 1 h. After this recording, baseline activity was obtained by recording the pups in isolation for 15 min (Fig. 2*A*). LFP low-frequency band oscillations (δ, θ, and β bands) were normalized to baseline and analyzed as they appear to be critical in long-range functional connectivity in early life (Del Rio-Bermudez et al., 2017).

We observed an overall tendency for increased LFP power across frequencies when the dam was in the nest compared to out of the nest (0–30 Hz; dam location: *F*_(1,24)_ = 3.261, *p* = 0.08, two-way RM ANOVA; Fig. 2*B*,*C*). When data were analyzed by specific oscillatory frequency bands (i.e., δ: 0–3.9 Hz; θ: 3.9–7.8 Hz; and β: 15.6–30 Hz), we observed a significant increase in power in δ (*t*_(12)_ = 2.580, *p* = 0.02, paired *t* test; Fig. 2*D*) during maternal presence compared to the out of the nest condition, as well as a trend in θ (*t*_(12)_ = 1.934, *p* = 0.08, paired *t* test; Fig. 2*E*) and no significant difference in β (*t*_(12)_ = 1.639, *p* = 0.13, paired *t* test; Fig. 2*F*; Table 1). The current results as well as a previous report (Sarro et al., 2014) highlight the relevance of lower frequency oscillations at an early age in response to maternal presence.

**Table 1.** Summary of statistical results

Figure	Statistical test used	Main effects	*Post hoc* (LSD test)
Figure 2*C* Across frequencies	Two-way RM ANOVA	Location: *F*_(1,24)_ = 3.261; *p* = 0.0835Frequency: *F*_(29,696)_ = 2.400; *p* < 0.0001Location × frequency: *F*_(29,696)_ = 0.6265; *p* = 0.9383	
Figure 2*D*, δ	Paired *t* test	*t*_(12)_ = 2.580, *p* = 0.02	
Figure 2*E*, θ	Paired *t* test	*t*_(12)_ = 1.934, *p* = 0.08	
Figure 2*F*, β	Paired *t* test	*t*_(12)_ = 1.639, *p* = 0.13	
Figure 3*C*, δ	Unpaired *t* test	*t*_(11)_ = 2.353, *p* = 0.04	
Figure 3*D*, θ	Unpaired *t* test	*t*_(11)_ = 1.264, *p* = 0.23	
Figure 3*E*, % time in nest	Unpaired *t* test	*t*_(11)_ = 0.09022, *p* = 0.9297	
Figure 3*F*, % time nipple-attached	Unpaired *t* test	*t*_(10)_ = 0.4373, *p* = 0.6712	
Figure 4*C*, δ	One-way ANOVA	*F*_(3,12)_ = 4.704, *p* = 0.0215	SAL-FLX vs SAL-SAL: *p* = 0.012SAL-FLX vs KET-FLX: *p* = 0.008SAL-FLX vs KET-SAL: *p* = 0.010SAL-SAL vs KET-FLX: *p* = 0.829SAL-SAL vs KET-SAL: *p* = 0.920KET-FLX vs KET-SAL: *p* = 0.908
Figure 4*E*, USVs	One-way ANOVA	*F*_(3,20)_ = 2.039, *p* = 0.1407	

SAL = saline, FLX = fluoxetine, KET = ketanserin.

### The effect of maternal presence on pups’ PFC activity is mediated through 5-HT2R

We next tested whether the serotonergic system contributes to the modulation of PFC activity in the pup in response to maternal presence. We used ketanserin to inhibit 5-HT2R. In each recording session, one pup was selected randomly and injected (intraperitoneally) with either vehicle (0.9% saline) or ketanserin (2.5 mg/kg). Fifteen minutes after injection, LFP was recorded for 1 h while simultaneously registering whether the dam was in or out of the nest (Fig. 3*A*). Data were expressed as a ratio of LFP power in/out of the nest*100. As shown in Figure 4*C*,*D* and described below, there was no effect of ketanserin on LFP activity during baseline (isolation).

When we analyzed the data within the oscillatory frequency bands of interest, we observed a significant reduction of the effect of maternal presence in ketanserin injected animals in the δ (*t*_(11)_ = 2.353, *p* = 0.04, unpaired *t* test; Fig. 3*B*,*C*) but not in θ band (*t*_(11)_ = 1.264, *p* = 0.23, unpaired *t* test; Fig. 3*B–D*). We verified that this difference was not related to changes in the dams’ behavior. To assess that, we measured the percentage of time the dam spent in the nest, as well as the number of times the dam entered the nest and we found no difference between the two groups (*t*_(11)_ = 0.09022, *p* = 0.9297, unpaired *t* test; Fig. 3*E*). Furthermore, in a separate set of animals, we tested whether the nursing behavior was affected in ketanserin injected pups. We found no differences in the time nipple-attached between saline and ketanserin injected animals (*t*_(10)_ = 0.4373, *p* = 0.6712, unpaired *t* test; Fig. 3*F*).

We thus demonstrated that blocking 5-HT2R inhibits the effect of maternal presence on the lowest LFP oscillatory frequency.

### Enhancing serotonergic transmission in isolated pups is sufficient to enhance LFP power in low-frequency bands compared to controls

In this last experiment, we tested whether an artificial increase of 5-HT is sufficient to increase low-frequency oscillation power in isolated pups compared to controls. To do so, pups were injected with the SSRI-fluoxetine (10 mg/kg). To test the specificity of fluoxetine action on serotonergic transmission, ketanserin was used to inhibit 5-HT2R (2.5 mg/kg). In the first part of this experiment, each individual pup was placed and recorded for 15 min in isolation in a 500-ml plastic beaker, then they were selected randomly to receive vehicle or ketanserin, injected, and placed back in the home cage. One hour later, each pup was selected pseudo-randomly to receive fluoxetine or vehicle. Following a wait time of 15 min, pups’ PFC activity was recorded for 30 min while they were isolated (Fig. 4*A*). This design resulted in four treatment groups: saline-saline; saline-fluoxetine; ketanserin-saline; and a ketanserin-fluoxetine (Fig. 4*A*).

When each frequency band of interest (δ and θ bands) was analyzed, we observed an increase in the saline-fluoxetine group in the δ (*F*_(3,12)_ = 4.704, *p* = 0.0215, one-way ANOVA; LSD *post hoc* test; Fig. 4*B*,*C*; Table 1) and θ bands compared to the other treatment groups (*F*_(3,12)_ = 3.668, *p* = 0.0439, one-way ANOVA; LSD *post hoc* test; Fig. 4*B–D*; Table 1). These data suggest that increasing serotonergic signaling, when pups are isolated from the dam, is sufficient to increase LFP low-frequency power compared to controls. Furthermore, this effect was completely abolished by 5-HT2R blockage further supporting that the effects of serotonergic neurotransmission on cortical LFPs in our model is mediated through 5-HT2R. Importantly, 5-HT2R antagonism alone did not change LFPs during baseline conditions (i.e., saline/saline vs ketanserin/saline did not differ significantly; Table 1). We thus demonstrated in isolated pups, that PFC activity in the low-frequency band can be artificially increased by increasing 5-HT compared to controls, an increase also observed when the dam was in the nest.

When separated from their mother and littermates, rat pups emit USVs in frequencies between 30 and 65 kHz, a range generally referred to as distress calls (Simola, 2015). These call have been found to be reduced by several antidepressant drugs, including fluoxetine (Simola, 2015). Consistent with the literature, although not statistically significant, we found a tendency for a reduction in the number of USVs in the fluoxetine treated group. Furthermore, ketanserin does not seem to block the reduction in USVs in fluoxetine treated pups and by itself tends to reduce the number USVs (*F*_(3,20)_ = 2.039, *p* = 0.1407, one-way ANOVA).

## Discussion

Neuronal activity during development shapes neuronal anatomy, connectivity and function. Extensive work has been done, especially in the visual system, establishing how environmental experience and neural oscillations during critical developmental periods affects neuronal development and function (Katz and Shatz, 1996). During early life, maternal behavior is a major source of environmental input for the pup. Models of maternal abuse/neglect have implicated poor maternal care as critical in the development of later-life cognitive and emotional deficits in the offspring, which has been supported by the human developmental literature and causally linked within the animal literature since the 1950s using a myriad of paradigms, including maternal separation, high/low licking of pups, and reduction of maternal resources for nest building (Hofer, 1994; Liu et al., 1997; Krugers et al., 2017; Walker et al., 2017). However, minimal research has focused on how maternal presence impacts the immediate response of the infant brain. By using a wireless LFP recording system that permitted undisturbed, direct, and simultaneous assessment of the effect of maternal presence on the brain activity of young pups, our results pinpoint maternal contact with pups in the nest as a major regulator of neuronal activity.

This study establishes a contribution of 5-HT in the regulation of pups’ cortical oscillations. Specifically, we demonstrated that maternal contact is correlated to an increase of low-frequency oscillations in the ACC region of the PFC, and this increase is mediated by 5-HT2R. Furthermore, we found that enhancing serotonergic transmission using a SSRI is sufficient to enhance LFP power in low-frequency bands in a pattern similar to the one obtained when the dam is in contact with the pup. Importantly, this regulation was also abolished by blocking 5-HT2R. Together, these results establish that the serotonergic system is part of the network involved in the modulation of pups’ brain activity in response to maternal presence.

### Influence of maternal presence/absence in the nest on offsprings’ brain activity: importance of low-frequency oscillations and the PFC

Maternal presence/absence can critically affect developing animals. Among other things, it modulates the release of stress hormones, changes USVs and provides olfacto-tactile inputs to the pups (Kuhn and Schanberg, 1998; Ise and Ohta, 2009; Hanganu-Opatz, 2010).

Relative to neuronal activity, we found that maternal presence in the nest increases LFP power in low-frequency bands in the PFC (Fig. 2). This increase of cortical LFP power can be related to various factors during the in nest period. Although, we did not monitor the specific activity of the dam in the nest, we can hypothesize, based on previous work, that this increase is related to (1) nipple-attachment, shown to induce an increased in low-frequency band in the somatosensory cortex (Sarro et al., 2014); (2) tactile stimulation arising from the mother/littermates, shown to drive somatosensory cortex activity (Yang et al., 2009; Akhmetshina et al., 2016a); (3) maternal olfactory stimulation (Moriceau and Sullivan, 2005, 2006) and most probably a combination of these and other factors.

Our results support the importance of LFP low-frequency band oscillations in young pups. Slow oscillatory activity is thought to be important in the coordination between long distance brain regions and is also proposed to be critical for development (Khazipov and Luhmann, 2006; Uhlhaas et al., 2010). Notably, in premature humans, using electroencephalography, rhythmic activity has been observed and δ oscillatory band has been described as the main oscillatory frequency band on top of which faster rhythms, namely δ brushes, can be superimposed (for review, see Khazipov and Luhmann, 2006). Interestingly, δ brushes have been proposed as biomarkers to detect neurocognitive deficits in human infants (Whitehead et al., 2017). In rodents, few studies have recorded neuronal activity in pups *in vivo* but in P1–P8 rat pups, Khazipov et al. (2004) demonstrated the emergence of spindles in somatosensory cortex related to sensori-motor integration thought to be analogous to δ brushes in humans. In addition, Adelsberger et al. (2005) also revealed, *in vivo*, an intrinsic neocortical calcium slow rhythmic activity in resting mouse pups. The effect of maternal presence we observed on LFP slow oscillation power might thus have critical effects on the developing brain.

The effect on the PFC is of high interest as it is a structure involved in a wide range of functions such as executive control or working memory (Miller and Cohen, 2001). As highlighted by Kolb et al. (2012), the PFC has an extended developmental period and early life experience, such as maternal care, can critically shape this development leading to long-term consequences in adulthood. Indeed, it has been shown that maternal separation leads to modification of the dendritic arborization in PFC pyramidal neurons (Monroy et al., 2010; González-Mariscal and Melo, 2017). In addition, Chugani et al. (2001) showed that institutionalized orphans display reduced glucose metabolism in prefrontal areas associated with neurocognitive deficits. Critically, it has also been shown that early maternal separation modifies the prefrontal-hippocampal network in rats recorded at P21–P22, especially in males (Reincke and Hanganu-Opatz, 2017). Notably, the authors show a decrease of LFP θ-β oscillatory band power in the prelimbic part of the PFC as well as a decrease of prelimbic neurons spikes phase-locking to θ oscillation recorded in the hippocampus. This study highlights the fact that a wide network of structures can be impacted by maternal interactions.

### Role of serotonin in the modulation of pups’ neural activity by maternal care

Young altricial animals rely on maternal regulation of their physiologic function and homeostasis through sensory stimuli received during maternal care, including somatosensory and chemosensory stimuli (Hofer, 1994). The influence of sensory stimulation on the infant’s immediate behavior and later-life neurobehavioral function has been documented by decades of research. This work has highlighted an important role for glucocorticoid levels (Levine, 1967; Meaney et al., 1988). Subsequent studies have shown that handling/licking/grooming regulate glucocorticoid receptor transcription via the serotonergic system (Mitchell et al., 1990, 1992; Laplante et al., 2002; Hellstrom et al., 2012). Interestingly, in rats, maternal separation decreases the 5-HIAA/5-HT ratio in PFC (Xue et al., 2013; González-Mariscal and Melo, 2017). Relative to the different 5HT receptor subtypes, it has notably been shown that animals subjected to maternal separation exhibit deregulation of 5-HT2R function (Benekareddy et al., 2010). Along with these lines of evidence, showing a contribution of serotonergic signaling to the effect of maternal presence on pups’ brain activity, we found, *in vivo* in behaving pups, that blocking serotonergic signaling via 5-HT2R abolished the increase in power in low-frequency bands observed when the dam was in the nest (Fig. 3). This effect was not due to a general lowering of LFP power due to the 5-HT2R antagonist ketanserin as we did not observe a change in cortical activity between saline and ketanserin injected animals (Fig. 4*C*,*D*). The effect of ketanserin we observed might be indirect and linked to a change of the dam’s behavior in regard to the injected pup (i.e., grooming, nursing) known to differentially modulate pup’s LFP activity (Sarro et al., 2014). We did not record the specific dam’s behaviors in the nest, however we did not find differences in the frequency and duration of time the dam spent in the nest, nor the time the pups spent nipple-attached (performed in a different set of animals), between ketanserin and saline injected groups.

In the future, it would be interesting to measure real-time release of 5-HT during maternal interaction using microdialysis or cyclic voltammetry. Unfortunately, these techniques, in the current state of the art, cannot be performed wirelessly, thus impairing analyses during active maternal-pup interaction.

Finally, we found that enhancing 5-HT signaling using a SSRI induces an increase of low LFP frequency band oscillatory power in isolated pups compared to controls. This effect may be correlated for example with changes in the pup’s behavior (e.g., USVs), or/and activity in other brain areas; for example, the SSRI citalopram was been shown to reduce the spontaneous, as well as the sensory evoked, activities in the barrel cortex of P2–P5 rat pups (Akhmetshina et al., 2016b). However, from our data, it does not seem that the modulation of cortical activity can only be ascribed to changes in USVs. Indeed and consistent with previous studies, we found a tendency for a reduction in the number of USVs in pups injected with fluoxetine (Simola, 2015). However, we found that ketanserin does not seem to block the reduction in USVs in fluoxetine treated pups and furthermore, as previously described, ketanserin by itself seems to reduce USVs (Schreiber et al., 1998). These data thus suggest a dissociation between USVs and LFP power in our treatment conditions.

The tight regulation of 5-HT levels during development is of extreme importance, with both low and high levels of serotonergic signaling seeming to be detrimental. Indeed, it appears that there is a critical period and an optimal range of 5-HT level and that any deviation from this range can alter normal brain development (Yu et al., 2014; Suri et al., 2015; Gingrich et al., 2017). For example, administration of the SSRI fluoxetine to rodent pups has been shown to lead to behavioral deficits in adulthood (Ansorge et al., 2004; Sarkar et al., 2014; Rebello et al., 2014). Interestingly, these behavioral deficits are avoided by concomitant administration of fluoxetine and ketanserin (Sarkar et al., 2014). Furthermore, treatment with the 5-HT2R antagonist, ketanserin, prevented the emergence of anxiety behaviors and changes in 5-HT2R expression associated with maternal separation (Benekareddy et al., 2011).

## Conclusion

In this report, we reveal that maternal presence modulates the pups’ cortical activity via the serotonergic system stressing the importance of maternal care in the activity of this tightly regulated system. 5-HT is one of the neuromodulators involved in the regulation of the offspring’s brain activity by maternal care, other neuromodulators and brain regions are probably jointly or in parallel involved such as the noradrenaline in the somatosensory cortex (Sarro et al., 2014) or the acetylcholine in the prefrontal-hippocampal network (Janiesch et al., 2011). Further studies will help us dissect the whole extent of this modulation.

## Synthesis

Reviewing Editor: Esther Stoeckli, University of Zurich Decisions are customarily a result of the Reviewing Editor and the peer reviewers coming together and discussing their recommendations until a consensus is reached. When revisions are invited, a fact-based synthesis statement explaining their decision and outlining what is needed to prepare a revision will be listed below. The following reviewer(s) agreed to reveal their identity: Eduardo Leonardo, Patricia Gaspar.

The revised version of the manuscript includes additional results and more experimental details. Thanks to these additions we agreed that the manuscript is acceptable for publication in its current form.
